# Evolution of embryonic development in nematodes

**DOI:** 10.1186/2041-9139-2-18

**Published:** 2011-09-20

**Authors:** Jens Schulze, Einhard Schierenberg

**Affiliations:** 1 University of Cologne, Biocenter, Zuelpicher Str. 47b 50967 Köln, Germany

**Keywords:** nematode, embryogenesis, cell lineage, polarity, symmetry formation, cell specification, evolution, *Tobrilus*, *Prionchulus*, *C. elegans*

## Abstract

**Background:**

Nematodes can be subdivided into basal Enoplea (clades 1 and 2) and more derived Chromadorea (clades 3 to 12). Embryogenesis of *Caenorhabditis elegans *(clade 9) has been analyzed in most detail. Their establishment of polarity and asymmetric cleavage requires the differential localization of PAR proteins. Earlier studies on selected other nematodes revealed that embryonic development of nematodes is more diverse than the essentially invariant development of *C. elegans *and the classic study object *Ascaris *had suggested. To obtain a more detailed picture of variations and evolutionary trends we compared embryonic cell lineages and pattern formation in embryos of all 12 nematode clades.

**Methods:**

The study was conducted using 4-D microscopy and 3-D modeling of developing embryos.

**Results:**

We found dramatic differences compared to *C. elegans *in Enoplea but also considerable variations among Chromadorea. We discovered 'Polarity Organizing Centers' (POCs) that orient cleavage spindles along the anterior-posterior axis in distinct cells over consecutive cell generations. The resulting lineally arranged blastomeres represent a starting point for the establishment of bilateral symmetry within individual lineages. We can discern six different early cleavage types and suggest that these variations are due to modifications in the activity of the POCs in conjunction with changes in the distribution of PAR proteins. In addition, our studies indicate that lineage complexity advanced considerably during evolution, that is we observe trends towards an increase of somatic founder cells, from monoclonal to polyclonal lineages and from a variable (position-dependent) to an invariable (lineage-dependent) way of cell fate specification. In contrast to the early phase of embryogenesis, the second half ('morphogenesis') appears similar in all studied nematodes. Comparison of early cleavage between the basal nematode *Tobrilus stefanskii *and the tardigrade *Hypsibius dujardini *revealed surprising similarities indicating that the presence of POCs is not restricted to nematode embryos.

**Conclusions:**

The pattern of cleavage, spatial arrangement and differentiation of cells diverged dramatically during the history of the phylum Nematoda without corresponding changes in the phenotype. While in all studied representatives the same distinctive developmental steps need to be taken, cell behavior leading to these is not conserved.

## Background

**Figure 1:**
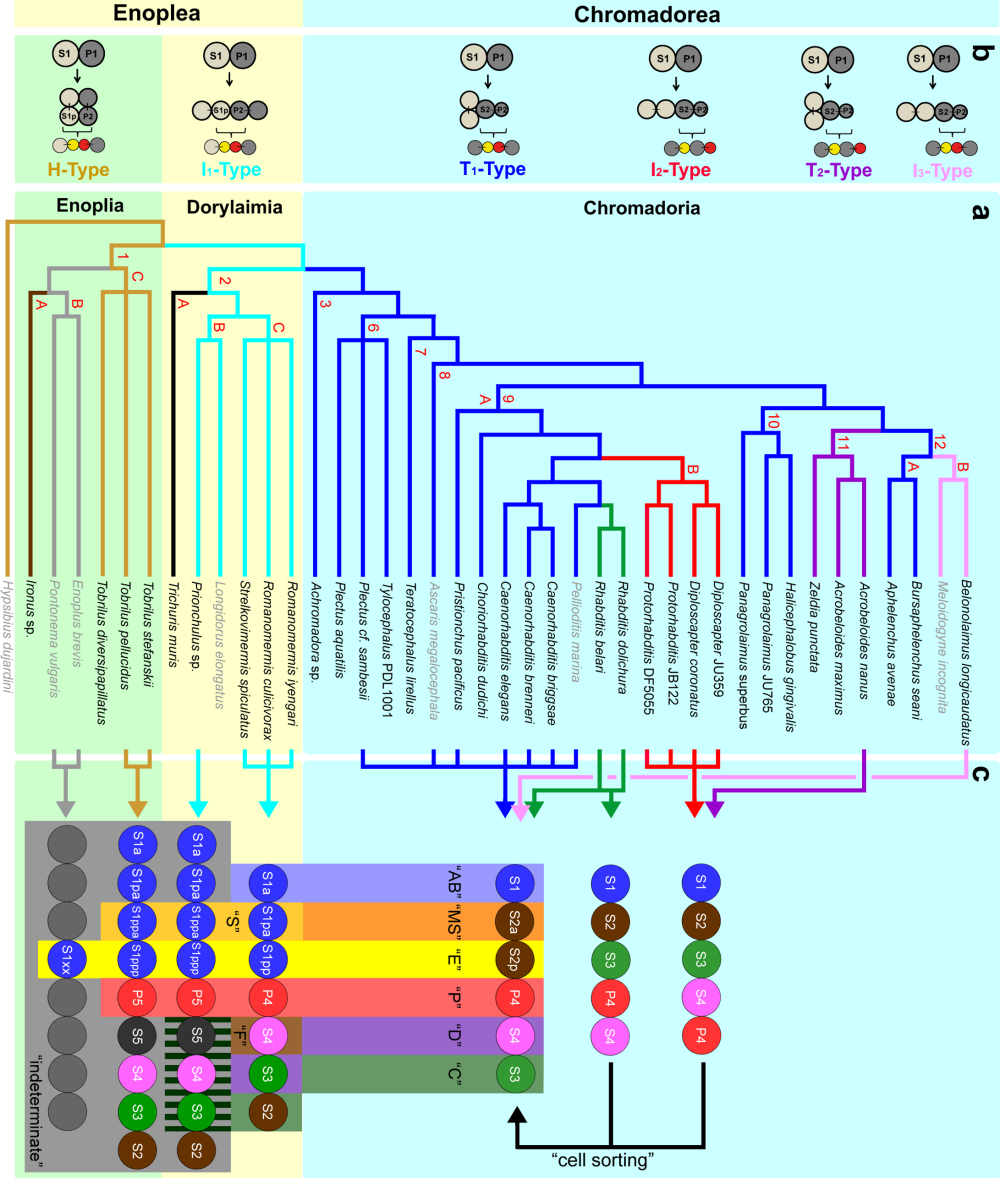
**Phylogeny and development**. **a**. Phylogenetic tree of nematodes (after [5]; clade 9 after [4]) with species whose embryogenesis is compared here. Different colors of lineage branches indicate distinct early cleavage patterns as indicated in Figure 1b. Individual clades (1 to 12) are marked with red numbers. Clades 4 and 5 are not included due to fragmentary data (see results). Based on distinct developmental characters described in the text some clades are further subdivided (A, B, C). Data of species written in grey have been extracted from the literature. References are given in the text. **b**. Six early cleavage types. Color of lettering corresponds to lineage branches where this pattern is found. Division of 2 to 4 cells (top) and origin and relative position of alimentary tract (Enoplea) or gut (Chromadorea) precursor (yellow) and germline cell P3 (red) generated with the next division are shown. **c**. Alignment of early blastomeres (colored circles) along the a-p axis. Colored columns indicate fate assignments ('AB' = AB-like and so on, for definition, see materials and methods). From clade 2C onward all early blastomeres can be assigned one of the six basic fates, however the position in the sequence of cells varies between species. Striated area indicates that in *Prionchulus *S3-S5 form largely bilaterally symmetric clones (for further description, see text). In some Chromadorea the initial order of founder cells is different to that in *C. elegans *due to the absence of PR in the germline. After cellular rearrangements ('cell sorting') they all merge into a single, standard pattern prior to the onset of gastrulation. PR, polarity reversal in the germline.

Over many decades various suggestions have been made concerning phylogenetic relationships among nematodes. With the availability of an increasing number of gene sequences the phylogeny of this phylum was put on a more objective basis and resulted in major revisions of previous classifications [1-4]. In this work we refer to the phylogeny of Holterman *et al. *[5], dividing the phylum Nematoda into 12 different clades (Figure 1a). We followed the proposal of De Ley and Blaxter [2] based on molecular and morphological criteria and subdivide nematodes into the two classes Enoplea (clades 1 and 2) and Chromadorea (clades 3 to 12). The former consists of two subclasses Enoplia (clade 1) and Dorylaimia (clade 2). Molecular and morphological data indicate that clade 1 comprises representatives closest to the common ancestor of nematodes while Chromadorea include phylogenetically more derived species [2,5,6].

Our current picture of embryonic development in nematodes is essentially shaped by the striking similarity between the classic model system *Ascaris megalocephala *(Clade 8; Figure 1a; [7,8]) and *Caenorhabditis elegans *(clade 9; [9]; http://www.wormbook.org). Work on *C. elegans *and its closer relatives has provided an initial insight into the recent evolution of embryonic and postembryonic development in clades 8 to 10 and discloses wide homologies in features, phenotypes and cell lineages [4,10-19]. Nevertheless, our understanding of the evolution of embryogenesis in the nematode phylum is still fragmentary. Species studied so far were usually chosen because of easy accessibility and amenable breeding conditions, and therefore represent a biased minority of the taxon. But generalizations from developmental characters of model organisms have to be taken with caution, because these organisms are often highly derived [20].

Embryonic studies revealed distinct developmental characters of nematode species and higher taxa that can be related to their phylogenetic position [14,21-26]. But not only inter-species but also intra-species variations have been uncovered, for example plasticity in pattern formation in *Acrobeloides nanus *[21] and *Diploscapter coronatus *[27] or postembryonic mouth dimorphism in *Pristionchus pacificus *(clade 9; [28,29]). A high regulative potential was demonstrated by the hierarchy of somatic cell fate transformations after cell ablation in the early embryo of *A. nanus *(clade 11; [30]). Even more dramatic peculiarities are found in Enoplea. Asymmetric cleavages and distinct cell lineages are initially missing in clade 1B (*Enoplus brevis, Pontonema vulgaris*) and only a gut lineage is present [22,31]. Development of *Tobrilus diversipapillatus *(clade 1C) is characterized by a prominent coeloblastula [32], a developmental character thought to be absent in nematodes. Compared to *C. elegans, Romanomermis culicivorax *(clade 2C) displays major differences in the establishment of embryonic polarity, pattern formation, programming of somatic founders and cell lineage complexity [33,34]. Hence, embryonic development of nematodes is much more diverse than the essentially invariant development of *C. elegans *and its closer relatives indicates.

Although in the Enoplea, development is usually much slower than in *C. elegans*, and their embryos are less transparent, we performed an extended analysis of development in selected species of this poorly studied basal group. Additionally we studied development in more detail in those clades of Chromadorea where only limited embryonic data have been available so far (Figure 1a). With these results, we now can address to what extent (i) nematodes follow a common general developmental program to generate the body plan typical for this phylum and (ii) developmental differences can be related to phylogenetic position (that is whether specific points can be defined where during evolution certain characters first appeared). The question which type of early cleavage was followed by the last common ancestor of nematodes has been controversially discussed in the past [35,36]. Therefore, we explored the notion that a nematode species with invariant polyclonal lineages generated by a fixed set of founder cells, such as *C. elegans *may have evolved from an ancestor such as *Enoplus *with just a single monoclonal lineage and a predominantly variable early embryogenesis.

## Methods

### Strains and culture

The strains *Acrobeloides maximus *(DF5048), *Acrobeloides nanus *(ES501), *Caenorhabditis brenneri *(SB280), *Caenorhabditis briggsae *(AF16), *Caenorhabditis elegans *(N2), *Choriorhabditis dudichi *(SB122), *Diploscapter *sp. (JU359), *Diploscapter coronatus *(PDL0010), *Halicephalobus gingivalis *(JB128), *Panagrolaimus *sp. (JU765), *Panagrolaimus superbus *(DF5050), *Plectus aquatilis *(PDL0018), *Plectus cf. sambesii *(ES601), *Pristionchulus pacificus *(PS312), *Protorhabditis *sp. (DF5055), *Protorhabditis *sp. (JB122), *Rhabditis belari *(ES103), *Rhabditis dolichura *(ES101), *Teratocephalus lirellus *(JB049), *Tylocephalus *sp. (PDL1001), *Zeldia punctata *(PDL0003) are cultured at 23°C on minimal agar plates essentially as described in [26]. Gravid animals of *Achromadora sp*., *Aphelenchus avenae, Bursaphelenchus seani, Ironus sp*., *Prionchulus sp*., *Tobrilus diversipapillatus, Tobrilus pellucidus *and *Tobrilus stefanskii *are isolated from soil samples of various origins essentially as described in [21]. *Romanomermis culicivorax, Romanomermis iyengari *and *Strelkovimermis spiculatus *were kindly provided by Dr. Edward Platzer, University of California, Riverside, CA, USA. *Trichuris muris *was kindly provided by Dr. Heinz Mehlhorn, Heinrich-Heine University, Düsseldorf, Germany. *Belonolaimus longicaudatus *was analyzed in the laboratory of Dr. Ole Becker, University of California, Riverside, CA, USA. Some gravid *Enoplus brevis *were kindly provided by Dr. V. Malakhov, Moscow State University, Russia, others were isolated from salt marsh soil supplied by Dr. W. Armonies, AWI List, Germany. Additional strains were obtained from Paul de Ley and Jim Baldwin, University of California, Riverside, CA, USA; Marie-Anne Felix, University Jacques Monod, Paris, France; Wouter Houthoofd and Wim Bert, University of Ghent, Belgium; Walter Sudhaus, Freie Universität Berlin, Germany; and Ralf Sommer, Max-Planck-Institute for Developmental Biology, Tübingen, Germany.

### Cell nomenclature and cell fate assignments

Projection of the *C. elegans *standard cell nomenclature [9,37] onto other nematodes implies the presence of similar cell differentiation patterns, which is not necessarily true. Therefore, we apply neutral lineage names (S1-S4/S5, somatic founder cells; P1-P4/P5, germline). We use the standard nomenclature (AB, MS, C, D) to indicate cell fates that correspond to those in *C. elegans *as described in [34]. In this species but not necessarily in other nematodes S1 = AB, S2 = EMS, S3 = C, S4 = D.

Although we traced cell divisions only up to a few hundred cells, we could assert the degree of variation from the pattern found in *C. elegans *(fixed lineages with unambiguous fate assignments). As we did not observe prominent rearrangements of cell clones after gastrulation we were able to assign fate categories ('AB'-like, 'S'-like, and so on; Figure 1c) to each of the founder cells based on position, structure and behavior of their descendants [34]. These cell fate categories imply similarities to *C. elegans *in terms of cell types derived from this lineage (for example 'AB', generates at least the majority of neurons; 'S' contributes to the pharynx) but may differ in detail. 'E' stands exclusively for gut fate in all studied species.

### Microscopy

With the exception of Enoplus (see below) 1-cell and 2-cell stage embryos collected from culture plates or cut out of gravid adults were mounted on slides carrying a thin 3% agarose layer as a mechanical cushion. The cover slip was sealed with melted petroleum jelly. Embryonic development was studied with DIC optics using a 100 × objective. Stacks of optical sections were digitally recorded at 30 to 60 second intervals and at 23°C with a 4-D microscope (Zeiss, Axioscope 2 mot). 3-D tracing of cell behavior and generation of cell lineages were software-supported (Simi Biocell; Unterschleißheim, Germany) essentially as described in [34]. Due to a sticky surface *Enoplus *1-cell stages adhered reliably to an untreated microscope slide. This was immersed into a petri dish filled with brackish water (ratio sea water to tap water, 2:1) and then covered with aluminum foil. Development was recorded on time lapse video tape with a 20 × water immersion objective. Evaporated water was replaced daily. If not stated otherwise the development of at least three embryos per species has been analyzed.

## Results

### Embryogenesis of *Enoplus brevis*

**Figure 2:**
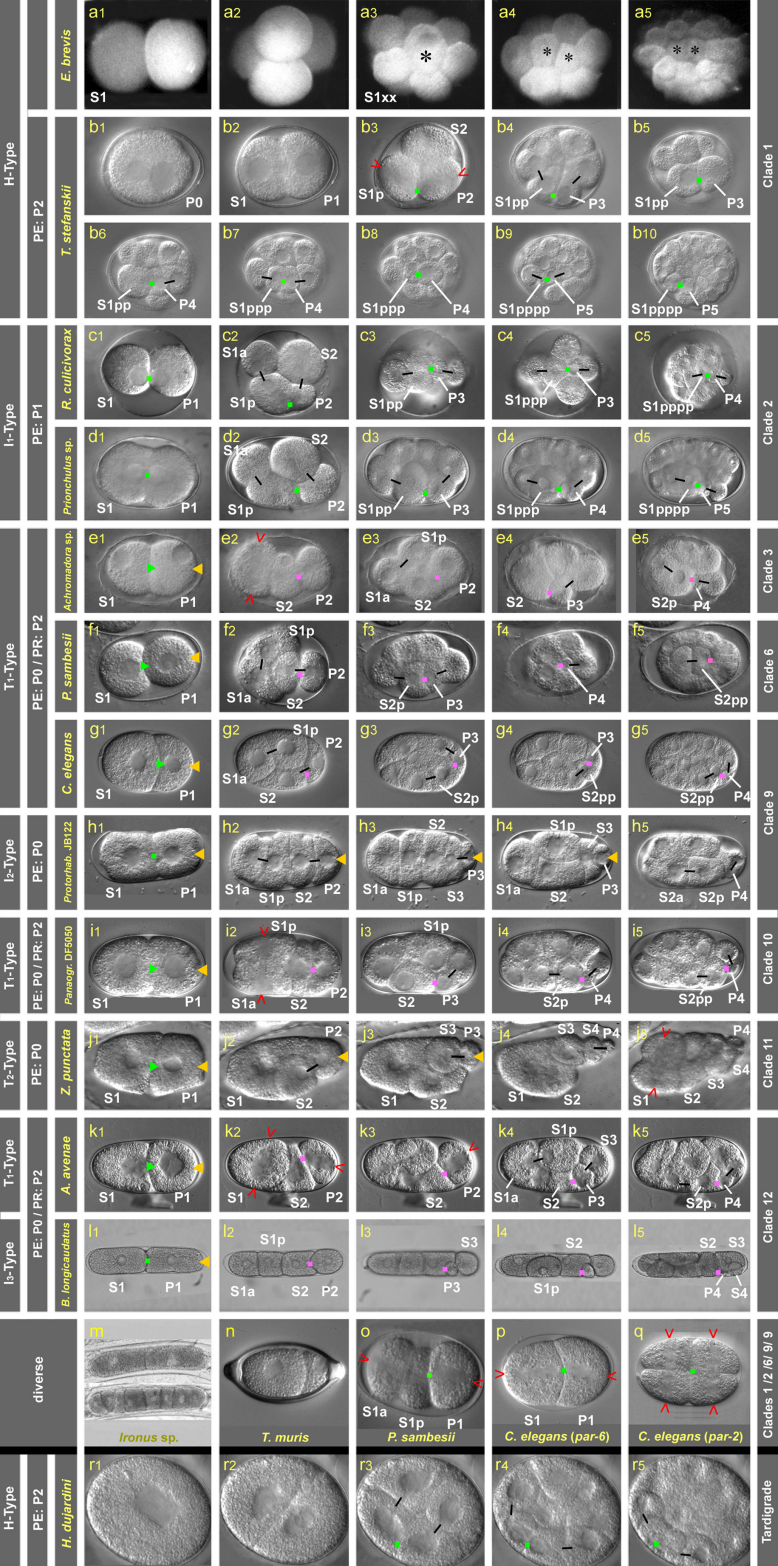
**Cleavage and polarity**. Early cleavage patterns in 12 nematode species (**a**-**l**) and the tardigrade *Hybsibius dujardini *(**r**). In addition, single images of selected nematode embryos are shown (**m**-**q)**. Representatives are ordered according to clades (right margin). On the left margin the type of early cleavage, stage when visible polarity is established (PE) and polarity reversal in the germline (PR) are indicated where applicable. Position and action of POCs deduced from cell behavior are marked. Green dot, primary POC acting on two adjacent cells; green arrowhead, primary POC acting on P1 only; purple dot, secondary POC acting on two adjacent cells; yellow arrowhead, tertiary POC, acting on germline cells; open red arrowhead, orientation of cleavage spindle; black bars connect sister cells. Figure 2r was taken from [48] with permission from Elsevier; Figure 2q was taken from [67].

We confirmed, with photo documentation, the observations of Russian researchers (see introduction). First cleavages are equal (Figure 2a1-a2) and lead to variable spatial arrangements. In the 8-cell stage one blastomere divides after a delay (Figure 2a3, black asterisk). This cell is the founder of the gut lineage. During gastrulation its two daughter cells are translocated into the interior of the embryo (Figure 2a4-a5) as in *C. elegans*.

### Embryogenesis of *Tobrilus stefanskii*

While in the early *E. brevis *embryo, only a single fixed cell lineage exists [31], in *Romanomermis culicivorax *already six fixed lineages are present as in *C. elegans *even though with different fate assignments [34]. Searching for species occupying intermediate evolutionary steps between these two extremes, we analyzed early embryogenesis of the basal nematode *Tobrilus stefanskii *(n = 11; clade 1C). With its early symmetric cleavages, the absence of visible cell lineages and its prominent coeloblastula, first described for another member of the genus [32], embryogenesis differs strikingly from *C. elegans *(clade 9A; [9]) and *R. culicivorax *(clade 2C; [33,34]).

*T. stefanskii *is a gonochoristic species with oviposition at the 1-cell stage preceding pronuclear fusion (Figure 2b1). The first division generates cells of equal size (Figure 2b2). Both spindles in the 2-cell stage orient at right angles to the first division, following the 'centriolic principle' (that is during consecutive cleavages spindles form at right angles to each other due to changing positions of centrioles; [38,39]). We named this early cleavage pattern 'H-Type' (Figure 1b). The first two cell division rounds produce descendants of equal size (Figure 2b2-b3). In the 4-cell stage various spatial arrangements are found [22,32]. Then the first asymmetric cleavages are observed. Based on these and subsequent asymmetries, and on specific cell behavior and lineage analysis described below, cells could be assigned lineage names in retrospect (for cell nomenclature, see materials and methods). One blastomere of the 4-cell stage shows the stem cell behavior and additional features typical for a germline cell in other nematodes (for example permanent contact with gut precursors; see below). Hence, we named it P2 and its sister S2. The remaining two cells are then daughters of S1. For better comparison with other species we named them S1a and S1p, although position relative to each other is variable. The nuclei in the cell cousins S1p and P2 leave their central position and become located side by side at the cell membrane (not shown). This is as found in *R. culicivorax *where, however, cell asymmetry starts to form already in the 2-cell stage (Figure 2c1; [33]). With the onset of division, the forming mitotic spindles in S1p and P2 become oriented and asymmetrically displaced toward a common point (green square, Figure 2b3). The orientation of these two spindles corresponds to the anterior-posterior (a-p) axis of the embryo. The resulting P3 is much smaller than its sibling S3, while size differences between S1pa and S1pp are less prominent (Figure 2b4).

**Figure 3:**
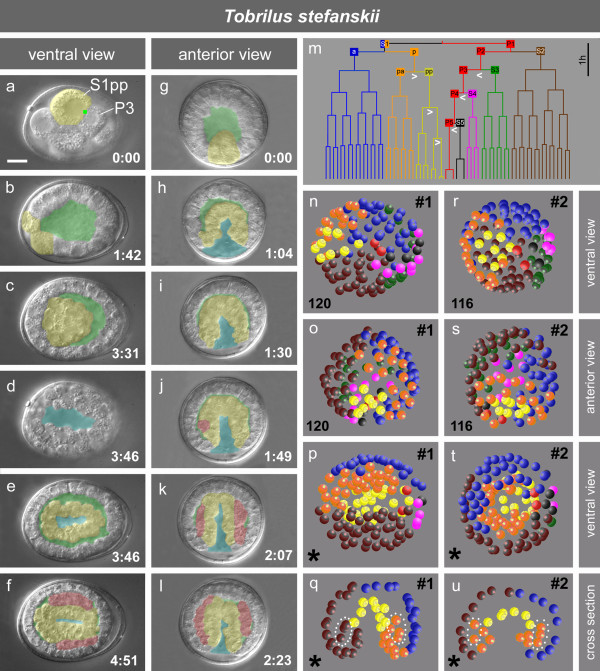
**Embryogenesis of *Tobrilus stefanskii***. **a-f; g-l**, cleavage, blastocoel formation, gastrulation and tissue formation of two embryos. Yellow, alimentary tract precursor(s); green, blastocoel; blue, slit-like blastopore; red, body muscle cells, green dot, primary POC; time in hours and minutes; **m**, cell lineage with seven somatic lineage branches and the germline (P0-P5); white arrowheads, asymmetric divisions; **n-q **and **r-u**, 3-D reconstructions of two embryos with similar stages indicating different spatial arrangements of cells. Each sphere represents a nucleus, color code as shown in m; p, t, partial reconstruction (*), not all cells could be traced, total cell number > 200; q, u, same stages as p and t, respectively, optical cross section; dotted areas, cells contributing to mesoderm. Scale bar, 10 μm.

**Figure 4:**
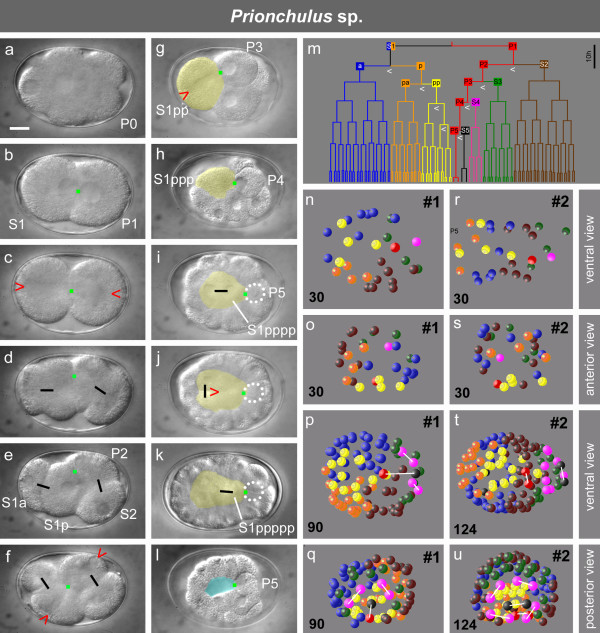
**Embryogenesis of *Prionchulus *sp**. **a-l**, cleavage, gastrulation and blastopore formation. Yellow, alimentary tract precursor(s); blue, slit-like blastopore; dotted circle, position of P5 (out of focus); green dot, primary POC; red arrowhead, orientation of cleavage spindle; black bars connect sister cells; **m**, cell lineage with seven somatic lineage branches and a germline (P0-P5); white arrowheads, asymmetric divisions; **n-q **and **r-u**, 3-D reconstructions of two embryos; p-q, t-u, differences in cell numbers (lower left corner) intentionally to visualize +/- bilateral symmetry in descendants of S4-S5 and to a lesser degree also of S3 in contrast to variable spatial arrangements of S1 and S2 descendants. Sister cells marked with white bars. Each sphere represents a nucleus; color code as shown in m. Scale bar, 10 μm.

**Figure 5:**
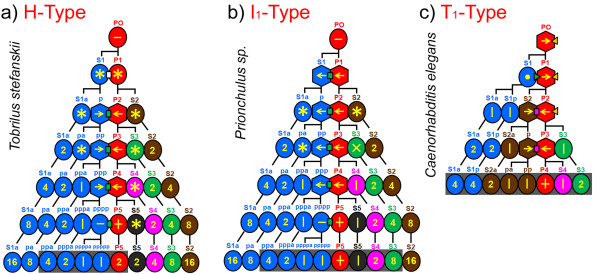
**Early cleavage and model of POC action**. **a-c**, Simplified early lineage trees of three nematode species with different cleavage types. A series of unequal cleavages attributed to the action of POCs results in cells arranged in tandem along the a-p axis. For symbols and color codes of POCs, see Figure 2. Note that cells performing l-r divisions belong to different generations. These cells or their descendants are shown in grey boxes to indicate lineage branches involved in the establishment of bilateral symmetry. Circles, blastomeres performing symmetric cleavages; hexagons, blastomeres performing asymmetric cleavages. Orientation of cleavage spindles: asterisk, variable; arrow, longitudinal, pointing toward smaller daughter cell; horizontal bar, longitudinal with daughters of equal size, vertical bar, left-right; circle, dorsal-ventral; 'X' somewhat variable generating imperfect symmetric clones; '**+**', germline cell giving rise to two mirror image gonadal arms in the anterior and posterior half of the adult.

The events described above are repeated when the cousins S1pp and P3 (Figure 2b4 to b6) and S1ppp and P4 (Figure 2b7 to b9) cleave asymmetrically (marked in Figure 3m). Spindles in S1pppp and P5 are oriented along the a-p axis as well but the divisions of these cells are not obviously asymmetric. The soma/germline cell pairs named above remain firmly attached to each other and their mitotic spindle microtubules appear to find distinct anchorage sites in the area of cell attachment (Figure 2b3 to b10) leading to a linear orientation of spindles. Consequently an array of cells forms along the future midline as a prerequisite for the establishment of bilateral symmetry (see paragraph below). This strongly resembles cell behavior in the early embryo of *R. culicivorax *(Figure 2c1 to c5; [33]) induced by the 'Region of First Midbody' (RFM). As we do not know whether the underlying mechanism is the same in both species we named it more generally 'Polarity Organizing Center' (POC). To distinguish it from other centers found in the higher-numbered clades (see below) we call it 'primary POC' (marked in green in Figures 2 to 5).

After having identified a germline, we traced the fate of the remaining cells. In the 8-cell stage S1pp and P3 are neighbors with nuclei asymmetrically positioned adjacent to the primary POC (Figure 3a), and both divide with delay (Figure 3m). S1pp turns out to give rise to the alimentary tract (that is gut+pharynx; Figure 3a to 3f). This is different from *R. culicivorax *[34] and *Enoplus *[31,40], where the founder cell for the alimentary tract is already present one cell generation earlier (Figure 1c). In Chromadorea the differences are more dramatic as there the gut is generated by S2p (Figure 1c and below; [7,9,21,22]).

As in other nematodes [21,41] the germline in *T. stefanskii *remains in permanent contact with the gut founder and its descendants. In contrast to *C. elegans*, in *T. stefanskii *P4 performs another asymmetric division resulting in S5 and P5 (Figure 2b10). The latter behaves like P4 in *C. elegans*, in that it divides symmetrically. Its daughters follow the gut precursors into the center of the embryo (Figure 3p, t).

Typical for all three of the *Tobrilus *species we looked at, is the formation of a prominent coeloblastula (Figure 3b). They undergo a 'canonical' gastrulation, with the invagination of a layer of alimentary tract precursor cells (Figure 3c, g to j, n to 3u; [32]). The blastocoel starts to form as early as the 4-cell stage and grows to its full size before the 100-cell stage. Gastrulation starts with the invagination of the eight S1pp descendants (Figure 3g, o, s). Other cell groups join them but their lineage origin varies among the analyzed embryos. As divisions continue, the immigrated cells fill the blastocoel (Figure 3c, g to 3i), leaving an oval-shaped blastopore furrow on the ventral side (Figure 3d) that reaches deep into the alimentary tract primordium (Figure 3k). Eventually it closes from the center in both directions along the a-p axis like a zipper, resulting in future mouth and anus at its ends (not shown) similar to *R.culicivorax *and other basal nematodes [22,34]. After immigration of future alimentary tract cells (leaving only small remnants of the blastocoel), body muscle precursors of various lineage origin move between these and the outer layer of cells (Figure 3f, j to 3l).

Lineage analysis and 3-D reconstructions (n = 9) revealed that in contrast to members of clade 1B in the early *T. stefanskii *embryo, three distinct cell lineages can be defined (Figure 1c), for gut ('E'), pharynx ('S'; additional cells contribute variably to this organ) and germline ('P'). For the remainder no fixed correlation between lineage and fate exists, that is the spatial arrangement of these blastomeres and their contributions to tissues is variable. This is exemplified in the two embryos shown in Figure 3. In embryo #1 (Figure 3p) descendants of S1pa (orange; left side) and S2 (brown; right side) form separate clones. Cells of both origins migrate in (Figure 3q) to contribute to the mesoderm. In embryo #2 (Figure 3t) S1pa descendants are located on both sides and at this stage cells between the inner and outer cell layer are all members of this lineage branch (Figure 3u). Among the other specimens of *T. stefanskii *we found various combinations of S1a, S1p and S2 lineages contributing to body muscle cells.

### Embryogenesis of *Prionchulus *sp

To narrow this gap between *Tobrilus *with three and *Romanomermis *with six lineages we studied a second representative of clade 2B, *Prionchulus *sp. (Mononchida, n = 4). In earlier studies contradictory conclusions had been drawn concerning the existence of early asymmetric cleavages and distinct cell lineages in this genus [42,43].

We found several basic similarities to *R. culicivorax *(Figure 2c1 to c5; [33,34]) in that the first division in *Prionchulus *is symmetric, nuclei of the 2-cell stage occupy adjacent positions (Figures 2d1, 4b), and both spindles become oriented along the a-p axis (Figure 4c to 4d). The latter indicates the action of a primary POC already in the 2-cell stage. We call this the 'I-Type' of cleavage. As it differs from similar I-Type cleavage patterns seen in higher-numbered clades (see below) we named it 'I_1_-Type (Figure 1b). In *Prionchulus*, S1p is definitely larger than S1a while size differences between S2 and P2 are less prominent (Figures 2d2, 4e). A similar pattern can be identified in (*Longidorus elongatus *Figure 1a; [44])

Restricted by the limited space in the eggshell, 4-cell stage blastomeres arrange themselves in two alternative rhomboid variants, whereby either S2 or P2 occupy a position adjacent to S1a (Figure 2d2, n = 2; Figure 4f, n = 2). In both of these S1p and P2 touch each other and form lineally oriented spindles (Figure 4f) resulting in daughter blastomeres arranged in tandem (Figure 2d3). The same process is repeated with the division of the cell pairs S1pp-P3 (Figures 2d3 to d4, g to 4h) and S1ppp-P4 (Figures 2d4 to d5, 4h to 4i). The fact that members of each pair do not move relative to each other underlines the continuous presence of a centrally located POC.

Gastrulation starts with the immigration of S1pppa+S1pppp in the absence of a coeloblastula (Figures 2d5, 4i). Descendants of S1ppa follow somewhat later and contribute to the pharynx. Thus, they show some resemblance to the behavior of MS (S2a) in *C. elegans*. However, as we could not find any contribution to body muscles we marked their fate with 'S' in Figure 1c. The same applies to *Tobrilus *(see above) and *Romanomermis *[34]. Contacts remain between P5, still positioned on the surface, and S1pppp and its posterior daughter S1ppppp (Figure 4i to 4k). *Prionchulus *shows high similarities to *Tobrilus *with respect to blastopore formation (Figure 5l) and the behavior of S1a, S1pa and S2 descendants. A further similarity, revealed by 3-D reconstructions of two embryos (Figure 4n to u) is the considerable positional variability of blastomeres.

As in *Tobrilus *we could only identify lineages for the alimentary tract ('S' + 'E') and the germline ('P'). However, here additional cells (S3-S5) occupy essentially invariant positions. They participate in the formation of bilaterally symmetric structures (Figures 1c, 4 p, q, t, u; see below) and appear to contribute to body muscles and hypodermis roughly corresponding to the C and D lineages in *C. elegans*. Due to limited transparency we could not ascertain whether S3-S5 make fixed contributions to the developing embryo. In any case these differ from the pattern found in *Romanomermis *and in *C. elegans*.

Embryogenesis in *Prionchulus *is much slower than that in *Tobrilus*. Initially, in both species cell cycles of all blastomeres are close to synchronous. Later, descendants of P3 and S1pp divide more slowly than other blastomeres. This is more obvious in *Tobrilus *than in *Prionchulus *(Figures 3m and 4m).

Our finding that cell lineages exist in the early *Prionchulus *embryo accords with the report of Drozdovskiy [42], however, we did not find the *Ascaris*-like invariant cleavage pattern with strict early bilateral symmetry and invariant cell positioning as shown in his sketches.

### Early embryogenesis in Chromadorea: 4 different cleavage types

#### T_1_-type, Clades 3, 4, 5, 6, 7, 8, 9A, 10, 12A (Figure 1b, blue lineage branches)

In the standard *C. elegans *(clade 9A; Figure 2g1 to g5), the first division is unequal, generating a larger S1 and a smaller P1 cell. While AB divides with transverse spindle orientation (following the centriolic principle; see above) P1 reorients its spindle to divide into a larger anterior S2 and a smaller posterior P2 (Figure 2g1 to g2). We named this early cleavage pattern 'T-typ'. In P2 a reversal of cleavage polarity (PR) takes place [41]. To distinguish this cleavage pattern we call it 'T_1_-type'.

In *Achromadora *(clade 3; Figure 2e1 to e5), the first division is more or less equal in size (Figure 2e1) otherwise early cell behavior is similar to *C. elegans*. Our limited observations in representatives of clades 4 and 5 (Desmodorida and Monhysterida) correspond well with descriptions by Malakhov [22] and make clear that, like *C. elegans*, early cleavages follow the T_1_-type pattern (data not shown). The same is true for species of clade 6 (*Plectus*; Figure 2f1 to f5 and *Tylocephalus*; [26]), clade 7 (*Teratocephalus*; [26]), clade 8 (*Ascaris*; [7]), clade 10 (*Panagrolaimus*; Figure 2i1 to i5) and clade 12A (*Aphelenchus*; Figure 2k1-k5). A slight variation of the T_1_-type cleavage pattern (green lineage branches) was detected in two representatives of the genus *Rhabditis*, where PR was found in either P2 or P3 [45].

#### I_2_-type, Clade 9B (Figure 1b, red lineage branches)

More prominent is a modification found in clade 9B with the genera *Protorhabditis *(Figure 2h1 to h5) and *Diploscapter *[27]. There, both first blastomeres divide with a-p oriented spindles resulting in a tandem arrangement of blastomeres [14] resembling the I_1_-type cleavage pattern found in *Romanomermis *and *Prionchulus *(Figures 2c1 to c5; d1 to d5). The elongate eggshell allows a strictly linear order of blastomeres after the divisions of S1, P1 and P2 (Figure 2h2, h3). The germline cells, due to the absence of PR, end up occupying positions posterior to their somatic sisters. To establish the d-v axis and to reach the typical neighborhood between the primordial germ cell P4 and the gut precursor S2p, prominent rearrangements ('cell sorting'; Figure 1c) are required (Figure 2h3 to h5) as described for *D. coronatus *[27].

#### T_2_-type, Clade 11 (Figure 1, purple lineage branches)

*Acrobeloides nanus *[21] and *Zeldia punctata *(Figure 2 j1-j5) follow the T-type of cleavage; however, in contrast to the T_1_-type PR is absent and therefore germline cells occupy the most posterior positions. Consequently, cell rearrangements among P2 descendants are required to reach a *C. elegans*-like pattern prior to the onset of gastrulation as described above for the 'I_2_-type'.

#### I_3_-type, Clade 12 (Figure 1, pink lineage branches)

*Belonolaimus longicaudatus *(Figure 2 l1 to l5) and *Meloidogyne incognita *[14] follow the I-Type of cleavage. However, this differs from the two 'I-types' introduced above (PR in P1 or absent) in that PR takes place in P2.

In summary, in nematodes we found six different early cleavage patterns, two among Enoplea and four among Chromadorea. In the phylogenetic tree (Figure 1a) I_2_-, T_2_- and I_3_-types are restricted to distinct branches indicating they are apomorphic modifications of the prevalent T_1_-type.

### The POC, a general developmental principle in nematodes?

Having detected the action of a POC in three representatives of Enoplea we wanted to determine whether such a mechanism is also characteristic for early embryogenesis of Chromadorea and thus may constitute a general developmental principle in nematodes.

In contrast to *Tobrilus, Prionchulus *and *Romanomermis*, three POCs can be defined in *C. elegans *which are involved in longitudinal spindle orientation and serial arrangement of founder cells. Laser ablation experiments in 2-cell embryos revealed a microtubule-pulling force at the anterior pole of P1 [46]. Located in the RFM, it resembles the 'primary POC' described above, although normally it only acts in P1 (Figure 2g1; green arrowhead). However, in embryos with defects in the expression of *par *genes, spindle orientations in P1 and S1 are altered (Figure 2p, q), suggesting an interaction between PAR proteins and the primary POC (see discussion). Another POC ('secondary POC'; marked in purple in Figure 2) positioned in the 'region of the second midbody' (RSM), initially orients spindles in the two sister cells S2 and P2 and subsequently in the cell cousins S2p+P3 and S2pp+P4 (Figure 2g2 to g5). This differs from the situation in Enoplea described above where S1, not S2, descendants are involved. For *C. elegans *it has been shown that the polarizing function of the secondary POC depends on the presence of MES-1/SRC-1 proteins (see discussion). A third POC ('tertiary POC'; marked in yellow in Figure 2) is established at the posterior pole of the 1-cell stage as a consequence of sperm entry [47]. It polarizes the fertilized egg, is crucial for asymmetric divisions of germline cells and depends on the polar distribution of PAR proteins (Figure 5c; see discussion).

As the majority of Chromadorea studied follow the T_1_-type of cleavage (Figure 1a; blue branches; Figure 2e to 2g, i, k) and early cell behavior is very similar to *C. elegans*, it indicates the activity of all three POC types. The other cleavage types (Figure 1b) can be explained with an altered expression pattern of PAR proteins affecting the function of the primary POC and the presence or absence of a PR in the germline (Figures 2, 5 and discussion).

### POC-based establishment of polarity beyond nematodes?

Our studies on early embryogenesis in nematodes revealed a characteristic cleavage pattern where, through several cell generations, two blastomeres orient their spindles towards a POC resulting in a series of asymmetric cleavages (Figure 2). We wondered whether this pattern is unique for nematodes and therefore compared it to early development of the tardigrade *Hybsibius dujardini *studied by Gabriel *et al. *[48]. According to the Ecdysozoa hypothesis [49] tardigrades are a sister group of nematodes. We found that early development of *Hybsibius *shows unexpected similarities to *Tobrilus *(Figure 2b). The first two divisions are equal with subsequent spindles perpendicular to each other (Figure 2r1 to r3), typical for the 'H-Type' of cleavage (Figure 1b). Then, tandem orientation of spindles in two neighboring cell cousins and subsequent asymmetric divisions indicate the presence of a POC (Figure 2r3, r4). As in *Tobrilus*, two adjacent cell cousins perform asymmetric, longitudinally oriented cleavages (Figure 2r5). Thus, a distinct POC-controlled division pattern is obviously shared between nematodes of clade 1C and at least one representative of tardigrades. In a species belonging to a different branch of tardigrades the H-type of early cleavage was observed, too, but not the reproducible unequal divisions [50].

### Establishment of bilateral symmetry

We found that despite considerable differences with respect to early development (for example H-, I-, T-cleavage types; PR in P1, P2, P3 or absent; Figures 1b, 2, 3m, 4m, 5), in all studied embryos with the exception of *Enoplus *(clade 1B; Figure 2a), a linear sequence of cells with different fates is generated along the a-p body axis due to the action of one or more POCs. Some or all of these divide into left and right descendants with equivalent fates on both sides of the midline. This way bilateral symmetry is established within individual cell lineages. To determine to what extent nematodes with different cleavage patterns vary in the way they develop bilateral symmetry, we compared early pattern formation in all 12 clades (Figure 1). Three representative species for the H-, I-, and T-types are shown in Figure 5.

In *T. stefanskii *(Figure 5a) a visible polarity is established in the 4cell stage (see above) and descendants of S1p and P2-P5 are aligned along the future midline. However, only three somatic cells (S1pppa, S1ppppa, S1ppppp) perform left-right oriented cleavages resulting in bilateral symmetric clones. Adhering to the centriolic principle (see above) the remaining blastomeres divide with variable spindle orientations giving rise to clones with indeterminate spatial positions.

In *Prionchulus *sp. (Figure 5b) in addition to three descendants of S1p also S4 and S5 execute divisions with transverse spindle orientation and subsequently generate bilaterally symmetric clones. S3 divides with oblique spindle orientation forming an imperfect early l-r symmetry (Figure 4p to 4u). The other cells show variable arrangements.

Thus, in *Tobrilus *and *Prionchulus *part of bilateral body symmetry must be established later, probably in a position-dependent manner as a result of cell-cell interactions.

In contrast, in *C. elegans *(Figure 5c) and all other studied members of clades 2C-12 all somatic founder cells or their early descendants form bilaterally symmetric clones (for modification of this principle in S1a cells, see [9]), independent of whether they follow the T_1_-(Figure 5c), T_2_-, I_2_- (Figure 5d) or I_3_-type of cleavage.

Comparing the three examples shown in Figure 5 a tendency can be observed towards an earlier and complete fixation of bilateral symmetry. The start of asymmetric divisions shifts from the 4-cell stage (*Tobrilus*) to the 1-cell stage (*C. elegans*) while the number of cell generations needed to perform all the l-r divisions described above decreases from seven (*Tobrilus*) to five (*C. elegans*) and the number of somatic lineages involved in early symmetry formation (Figure 1c) increases from two (*Tobrilus*) to five (*C. elegans*).

### Differences and similarities during ongoing embryogenesis

Our study makes clear that early embryogenesis differs dramatically among species, particularly within Enoplea. In addition, tissue formation varies during later stages. In a previous publication we showed that hypodermis is generated in *Romanomermis *very differently from that in *C. elegans *[34]. Preliminary data indicate that hypodermis formation in *Prionchulus *does not follow the pattern found in *Romanomermis*, in spite of both being members of the same clade. The peculiarities of gastrulation in *Tobrilus *(Figure 3) including the way cells assemble to form the gut are not only different compared to *C. elegans *but also to *Enoplus *(see above). We also found indications that founder cells contribute differently to the pharynx [9,34] and how this organ is formed (data not shown). However, eventually all variants seem to merge into a common pattern. The process of transforming a ball of cells consisting of three germ layers into an elongated worm during the "morphogenesis phase" starts with a ventral indentation separating head and tail regions in all studied nematodes. The progressive elongation of the embryo looks similar to *C. elegans *[51], although the degree of elongation varies considerably (that is juveniles may be longer and thinner). From this we conclude that the developmental constraints during the second half of embryogenesis are higher than during the early phase.

## Discussion

In this paper representatives of all 12 nematode clades (according to the phylogeny by [5]) have been compared with respect to their early embryogenesis. Our data document that the very similar and reproducible development found in the reference systems *Ascaris *and *C. elegans *(see introduction) exemplifies only one of many ways to generate a nematode worm from a 1-cell embryo. They indicate that the cleavage and differentiation program of blastomeres diverged dramatically during evolution. This is particularly obvious in Enoplia (clade 1) and Dorylaimia (clade 2). In each of these taxa developmental peculiarities and variations appear to be higher than in all Chromadorea (clades 3 to 12) combined. Because of distinct developmental features (Figures 1b, 2 to 5) it appears reasonable to break down clades 1, 2, 9 and 12 into subgroups (Figure 1a).

We believe that major developmental characters found in Enoplea but absent in Chromadorea (clades 3 to 12; Figures 1, 2) are plesiomorphic. These include the 'canonical' gastrulation in the genus *Tobrilus *with its large blastocoel (Figure 3; [32]) found in many other animal phyla including Nematomorpha (our unpublished results), the nearest phylogenetic neighbors of nematodes, and the similarity of early cleavage patterns between *Tobrilus *(Figure 2b) and the tardigrade *Hybsibius *(Figure 2r) which according to the Ecdysozoa hypothesis [49] belongs to a neighboring phylum. Thus, our findings support the positioning of Enoplea close to the base of the phylogenetic tree of nematodes postulated on grounds of molecular sequence data [2,5,6]. This implies that the route of lineage evolution went from an S1 to an S2 origin of the gut (Figure 1c; [22,40]), which required considerable modifications in cleavage pattern and fate assignment [34]. Furthermore, comparison of embryogenesis between nematodes, nematomorphs and tardigrades suggests that certain features (for example the absence of initial asymmetric cleavages) were shared by their last common ancestor.

### Variations, evolutionary trends and developmental system drift

Our data including those on *Romanomermis *(clade 2C) indicate a boost in lineage complexity, that is a stepwise increase from a single to five somatic lineages (Figures 1c, 5) and a change from a monoclonal to a polyclonal fate assignment [34]. This change, which appears to have coincided with the transition from Enoplea to Chromadorea (Figure 1), is correlated with an increase in early fate decisions, which in turn reduces the amount of cell migration necessary for proper tissue formation [12,34,52].

Comparing embryogenesis of representatives along the nematode phylogenetic tree (Figure 1a) we find that intra-species variation of early cell patterns decreases due to an increase of founder cells that generate descendants occupying fixed positions. Hence, our data are in favor of the conception that invariant development following distinct lineage programs is a derived and not an original feature (see introduction).

From the degree of individual variation within a species we can deduce how strongly fixed the developmental program must be and thus how a change in cleavage type (Figure 1b) may have been established during evolution. In *Plectus *(clade 6) we observed rare cases (2/< 100 embryos) where S1 cleaved with longitudinal (Figure 2o) rather than transverse spindle orientation (Figure 2f2), meaning a switch from a T_1_- to an I_2_-cleavage type. As this deviation was found to be compatible with normal embryogenesis it demonstrates that the developmental program must possess a sufficient degree of plasticity to allow the disregard of the 'centriolic principle' (see results section). Such an embryo must potentially cope with altered segregation of cytoplasmic components and changes in relative cell positions which in turn may affect inductive signaling. In addition, our observation suggests that the change from one to another cleavage type started with a modification that was initially rare in the population.

The divergence of developmental pathways without corresponding changes in the emerging phenotype ('developmental system drift'; [53]) seems to be a widely spread phenomenon in the animal kingdom. The present study gives additional examples for this. Moreover, it indicates that embryogenesis in nematodes includes general distinctive steps that need to be taken. However, cellular events leading to these are not conserved among species suggesting that evolutionary constraints have been low on cell behavior but high not only on structure and function of the juvenile but also on intermediate embryonic stopovers. Two examples may suffice to put our idea across. (i) The contact beween gut and germline (a critical feature in many systems; [54]) can be achieved via PR in either P1, P2 or P3 or alternatively via 'cell sorting' in species where PR is absent. (ii) Cell fate assignment can be reached in the absence of a fixed early cleavage program or alternatively via monoclonal or polyclonal cell lineages. Recent findings by Lin *et al. *[19] show that a similar relationship can be found between cells and molecules. In two closely related nematode species behavior of early blastomeres with alternative fates is identical while the underlying signaling network differs.

The enormous differences in genomes among even closely related species [55] in contrast to the conserved morphology indicate a particularly relaxed relationship between genotype and phenotype in nematodes. This discrepancy can be attributed to the special construction of nematodes including a single chamber hydroskeleton which allows adaptation to very diverse habitats but leaves little room for modifications of the body plan.

### Polarity organizing centers (POCs) and embryonic pattern formation

One of our central findings is the general presence of one or more POCs in early nematode embryos. While in the Enoplea *Romanomermis *[33], *Tobrilus *and *Prionchulus *(Figures 2b to 2d; 5a to 5b) we found evidence that early embryogenesis involves just a single (primary) POC, in Chromadorea up to three (primary, secondary and tertiary) POCs appear to be active in organizing orientation and asymmetry of divisions. Only for *C. elegans *do we have information about the molecular basis of the secondary and tertiary POC.

Laser ablation experiments in *C. elegans *[46] revealed a 'cortical pulling site' at the anterior pole of P1. Observations by Keating and White [56] support the view that the midbody between AB and P1 specifies a region of the cortex that directs rotational alignment of the centrosome-nucleus complex. This region of the first midbody (RFM; [33]) corresponds to our primary POC.

Most prominent is the activity of the tertiary POC at the posterior pole. Sperm entry initiates asymmetric distribution of PAR and LET-99 proteins [47,57-60] and subsequently anterior-posterior (a-p)-oriented spindles and asymmetric divisions in the germline [61,62]. However, in a member of clade 11 (Figure 1A) axis polarity was found to be independent of the sperm entry point [63].

Mutants and RNAi phenotypes of *pkc-3, par-2, par-3 *and *par-6 *in *C. elegans *demonstrate that a knockout of any of these genes changes the orientation of the cleavage spindle in the 2-cell stage and this way leads from the T-type to H- or I-types of cleavage (Figure 2p, q), even though followed by abnormal development [64-68].

To explain the different cleavage types in Chromadorea we propose that in contrast to Enoplea (where the primary POC always induces a-p spindle orientation in two adjacent cells; Figures 2b to d, 5a, b), the polarizing function in Chromadorea is controlled by PAR proteins [27]. This view is supported by mutations and RNAi experiments in *C. elegans *leading to abnormal cleavage patterns. The *par-2/par-3 *double mutant follows the I-type of cleavage, demonstrating that neither of these genes is required for longitudinal orientation of cleavage spindles [64]. According to our model the prevalent T-type (Figure 1b) can be explained with the anterior PAR complex suppressing the activity of the primary POC in S1. At the same time it gives a simple explanation why uniform distribution of anterior PAR proteins (after knockout of *par-2*) results in transverse spindle orientation in S1 and P1 (H-type; Figure 2q) and uniform distribution of posterior PAR proteins (after knockout of anterior *par *genes) leads to longitudinal spindle orientation in both cells (I-type; Figure 2p).

In *C. elegans *PAR proteins switch function after the 4-cell stage has been reached and are involved in the establishment of apical-basal polarity [69]. Instead the MES-1/SRC-1 system is required to continue a-p polarization of blastomeres [70-72] and to induce the reversal of cleavage polarity (PR) in P2 [41] as is the case in the T_1_- and I_3_-cleavage types (Figures 1b, 2). The expression domain of MES-1/SRC-1 corresponds to our secondary POC. Berkowitz and Strome [72] suggested that species without PR may have lost the MES-1/SRC-1 system (accordingly a secondary POC is not indicated in Figure 2h, j). However, the question then remains how a-p divisions in the germline can be maintained in the I_2_- and T_2_-types. A straightforward explanation could be a prolonged activity of posterior PAR proteins. Indeed, in *Protorhabditis *(clade 9B, Figure 2h) an extended posterior expression of PAR-1 in the germline was observed by Brauchle *et al. *[18].

The peculiarities of early development in those Enoplea that we followed in some detail (Figure 2a to d) gave no indications that *par *genes are involved in cell polarization along the a-p axis as found in *C. elegans*. Although it would appear that this function may have been newly acquired in Chromadorea, our fragmentary data from two other Enoplida indicate that the situation is less clear. *Trichuris muris *(clade 2A) performs an extremely asymmetric first division (Figure 2n; see also [22]), and in *Ironus *(clade 1A) three consecutive longitudinally oriented cleavages result in a tandem of eight cells (Figure 2m). Both patterns cannot be readily explained with the activity of a primary POC alone. As our preliminary analysis of the *R. culicivorax *(clade 2C) genome indicates that *par *genes are present it remains to be determined whether at least in some Enoplea they may play a role in establishing the primary body axis.

Consecutive asymmetric divisions following a POC-like principle are not restricted to invertebrates like nematodes and tardigrades but have been described for posterior blastomeres of the ascidian embryo as well [73]. In equivalent cells in the left and right half of the bilaterally symmetric embryo asymmetric positioning of cleavage spindles is induced by centrosome attracting bodies (CAB; [74,75]). The CAB contains proteins that are homologous to the anterior PAR proteins in *C. elegans *[76]. It remains to be determined whether this asymmetry generating mechanism can be traced back to the last common ancestor or has been acquired independently in all three taxa.

### Midline formation and bilateral symmetry

A bilaterally symmetric body is typical for all higher animals. For *C. elegans *it has been shown that this symmetry is generated early within individual lineages [9]. The construction of a bilateral body plan requires the presence of a midline, separating left from right. Our studies revealed that in all analyzed nematodes, except clade 1B, such a midline can be defined during early embryogenesis.

In contrast to the models proposed by Meinhardt [77] for planarians, insects and vertebrates, in nematodes bilateral symmetry is generated in a surprisingly simple way. Due to the actions of one or more POCs (described above) which cause longitudinal spindle orientations and cleavage asymmetries over consecutive rounds of division, an array of cells is formed along the a-p axis. Depending on the species, part or all of these divide into left and right daughters (Figure 5) from which bilaterally symmetric clones arise. It appears likely that such a strategy requires specific conditions, for example a low number of blastomeres and their early specification on a single cell basis, features typical for nematodes.

### Cell lineage and cell specification

The case of *C. elegans *demonstrates that even in a system with essentially invariant development and complex polyclonal lineages, inductive interactions are an integral part of the developmental program which requires specific cell contacts during narrow time windows [78-81]. The different early cleavage patterns described above (Figures 1b, 2) result in different cell neighborhoods. Either changes in cell contacts did not pose a problem during nematode evolution because interactions like those in *C. elegans *did not exist yet, or the network of interactions was adjusted simultaneously. Our data are in accordance with a stepwise establishment of such interactions going along with increasing invariance of cell positions. Experimental interference like recombining blastomeres [70,82] in basal representatives could give us a better idea to what extent the pattern and relevance of embryonic inductions changed during evolution. Analysis of cell-specification patterns and their molecular underpinnings, particularly in Enoplea, should help to discern between two alternative visions. (i) Inductive interactions in early nematode embryos are historic remnants. They reach back to times when fixed lineages where mainly absent (for example *Enoplus*; [31]) or only simple, monoclonal lineages existed (for example *Romanomermis*; [34]) and were required for diversification of cell fate. (ii) Alternatively, they are more recent acquisitions made possible after fixed lineages assured invariant cell neighborhoods.

## Conclusions

Nematodes are suitable objects to study evolution of development because species from all branches of the phylogenetic tree can be analyzed, embryos develop outside the mothers and most of them are transparent enough to perform cellular analysis *in vivo*. Our findings that early embryogenesis varies considerably among species indicates that constraints are high on the preservation of crucial developmental steps but not on cellular behavior leading to these. We argue that the direction of evolution went from indeterminate early cleavage without initial polarity to invariant development with establishment of polarity before division of the zygote. The observed action of a primary POC gives a clue how polarity in certain nematodes and other related taxa like tardigrades can be established in a way that differs from *C. elegans*, that is independent of the sperm entry point.

## Abbreviations

CAB: centrosome attracting bodies; POC: polarity organizing center; PR: reversal of cleavage polarity in the germline; RFM: region of first midbody; RSM: region of second midbody.

## Competing interests

The authors declare that they have no competing interests.

## Authors' contributions

Both authors contributed to the conception and design of the study, were involved in acquisition of data, its analysis and interpretation. Both authors drafted the manuscript and read and approved the final version.
